# Gender-specific genetic and epigenetic signatures in cardiovascular disease

**DOI:** 10.3389/fcvm.2024.1355980

**Published:** 2024-03-11

**Authors:** Justin Bridges, Jose A. Ramirez-Guerrero, Manuel Rosa-Garrido

**Affiliations:** Department of Biomedical Engineering, School of Medicine, School of Engineering, University of Alabama at Birmingham, Birmingham, AL, United States

**Keywords:** sex-differences, genetics, epigenetics, escapee genes, hi-C, chromatin structure

## Abstract

Cardiac sex differences represent a pertinent focus in pursuit of the long-awaited goal of personalized medicine. Despite evident disparities in the onset and progression of cardiac pathology between sexes, historical oversight has led to the neglect of gender-specific considerations in the treatment of patients. This oversight is attributed to a predominant focus on male samples and a lack of sex-based segregation in patient studies. Recognizing these sex differences is not only relevant to the treatment of cisgender individuals; it also holds paramount importance in addressing the healthcare needs of transgender patients, a demographic that is increasingly prominent in contemporary society. In response to these challenges, various agencies, including the National Institutes of Health, have actively directed their efforts toward advancing our comprehension of this phenomenon. Epigenetics has proven to play a crucial role in understanding sex differences in both healthy and disease states within the heart. This review presents a comprehensive overview of the physiological distinctions between males and females during the development of various cardiac pathologies, specifically focusing on unraveling the genetic and epigenetic mechanisms at play. Current findings related to distinct sex-chromosome compositions, the emergence of gender-biased genetic variations, and variations in hormonal profiles between sexes are highlighted. Additionally, the roles of DNA methylation, histone marks, and chromatin structure in mediating pathological sex differences are explored. To inspire further investigation into this crucial subject, we have conducted global analyses of various epigenetic features, leveraging data previously generated by the ENCODE project.

## 1 Introduction

Cardiovascular disease (CVD) stands as a predominant cause of morbidity and mortality across genders (1). However, notable distinctions characterize the manifestation, progression, and impact of this condition on males and females. Traditionally, heart failure has exhibited a higher prevalence in younger men compared to women, who tend to develop worse outcomes and higher mortality at older age. Through the lens of the physiology of CVD, coronary artery pathologies and heart attacks are more frequently associated with heart failure in males, while females often experience heart failure linked to hypertension, valvular heart disease, and heart muscle disorders.

In the early stages of life, the hearts of both sexes demonstrate remarkably similar sizes. However, a significant divergence emerges during puberty, with male hearts undergoing accelerated growth, resulting in a size difference that persists throughout life (2, 3). This divergence encompasses physiological, genetic, and epigenetic differences, each playing a crucial role in shaping how hearts respond to pathological stressors contributing to sex-specific cardiac disease patterns. When considering the factors influencing cardiac differences between males and females, two evident processes come to the forefront: the repercussions orchestrated by distinct sex chromosomes in both genders and the dynamic interplay of hormones between them. The study of processes associated with the sex chromosomes is progressing swiftly, thanks to the development of next-generation sequencing techniques. These advancements have facilitated the characterization of the transcriptional and genetic cardiac landscapes of males and females (4), revealing that genes evading X chromosome inactivation (escapee genes) and sex-biased genetic variations significantly contribute to differences in cardiac disease occurrence between the sexes. The second process involved in controlling cardiac sex differences relates to the varied hormonal landscape and its physiological implications. This area has undergone extensive study and experienced acceleration partially thanks to the development of the Four Core Genotypes mouse model. All these efforts unveil that women generally enjoy a favorable advantage in heart function, which gradually diminishes post-menopause, suggesting a crucial protective role of female hormones (estrogens) in shaping sex-related disparities under normal and pathological conditions (5).

**Figure 1:**
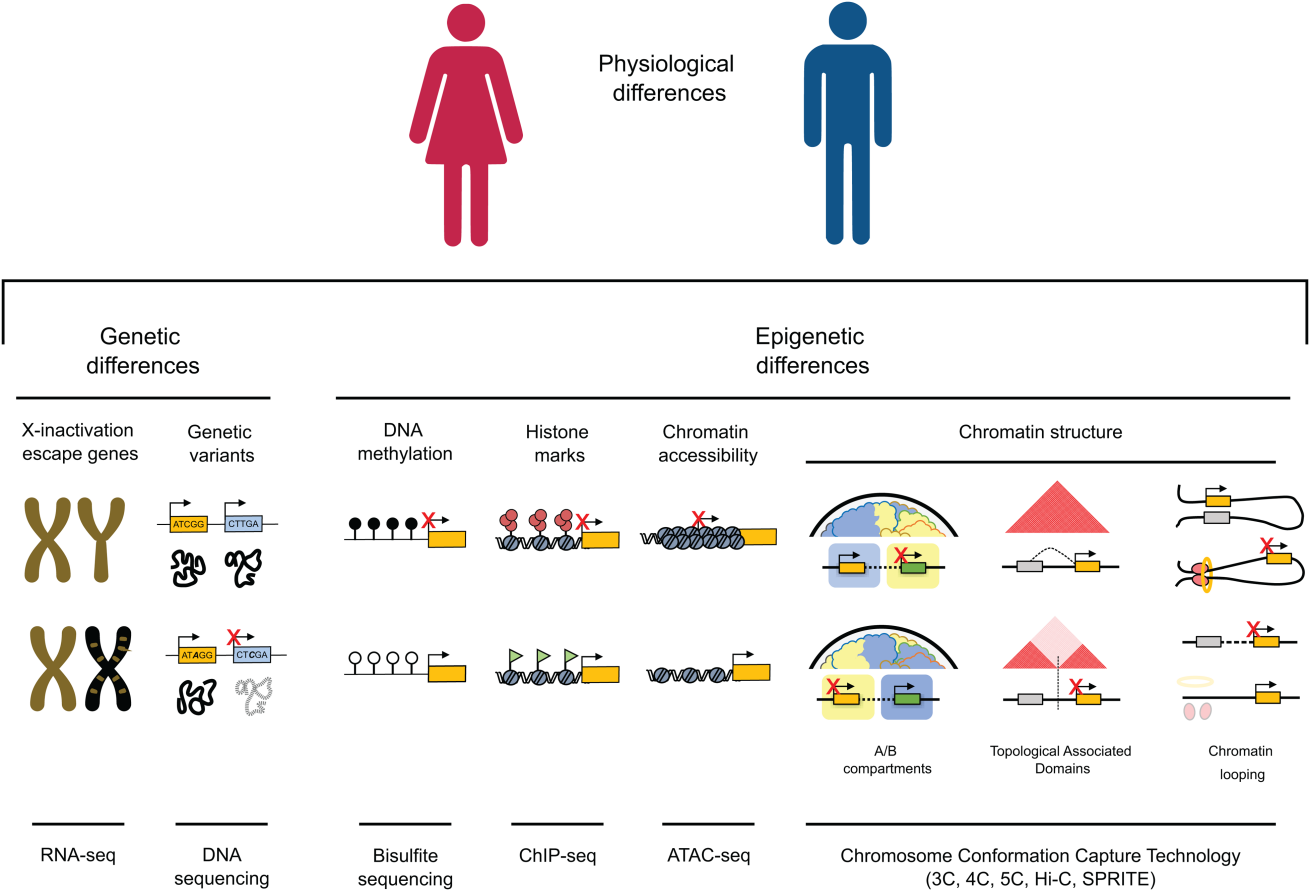
Genetic and epigenetic mechanisms contribute to gene expression variations between males and females. (1) Genes located on the X chromosome, which resist inactivation and are illustrated in gold on the inactivated chromosome in females (depicted in black), have the potential to influence cardiac dichotomies by disrupting the balance of gene expression. (2) Genetic variants can impact protein function and structure, potentially resulting in differences between sexes, especially in cases of homozygosity. (3) DNA methylation is a key player in sexual dimorphism, playing a crucial role in fostering sex differences in gene expression within specific genome regions. (4) The interplay of active and inactive histone modifications establishes and maintains sex-specific gene expression patterns that can result in differences between cardiac pathological phenotypes across males and females. (5) Chromatin accessibility, strongly influenced by the deposition of histone marks, can either enhance or suppress expression of genes associated with male or female characteristics, facilitating increased transcription of genes linked to a particular sex. (6) Higher-order chromatin structure plays a crucial role in governing gene expression across various scales. At a high level of organization, the positioning of a gene within an active (**A**) or inactive (**B**) epigenetic environment strongly influences its expression, leading to substantial variations in gene activity. On an intermediate scale, the different organization of Topologically Associating Domains (TADs), through the emergence, absence, or alterations in the strength of TAD boundaries, has the potential to reconfigure interactions between genes and regulatory elements. This rewiring can consequently lead to sex differences in gene expression. At the lowest levels of organization, the distinct arrangement of chromatin loops mediated by factors like CTCF and the cohesion complex (depicted as red circles and a yellow ring, respectively) can give rise to significant differences in gene isolation, relocation, and enhancer-promoter interactions, ultimately resulting in sex-specific gene expression.

A third, less obvious yet equally important factor in elucidating physiological distinctions between males and females is epigenetics (Figure 1). Coined by British developmental biologist Conrad Waddington in the 1940s, the term “epigenetics” encompasses all those changes capable of modifying gene expression without altering the DNA sequence that can be heritable and reversible. Over the last few decades, advances in techniques associated with DNA sequencing have fueled a surge in scientific interest and thus, research in epigenetics. Sex-specific differences in methylation patterns and chromatin accessibility have been identified in both healthy and tumor tissues (6) as well as during brain function (7). These studies demonstrate that expression of sex-biased genes is often regulated by sex-specific epigenetic mechanisms, underscoring the pivotal role of epigenetics in shaping male and female differences. In the realm of cardiac research, influence of epigenetic sex differences is an expanding field with important ramifications in DNA methylation, histone modifications and chromatin structure.

In this review, we provide an overview of primary disparities between sexes at the onset of cardiac pathology, with a specific focus on findings associated with genetic and epigenetic processes. To address existing knowledge gaps and contribute to a better understanding of certain epigenetic modifications, we leveraged data from the ENCODE project. Our aim is to shed light on the current state of knowledge and provide data that encourage and inspire further research in this critical area. By discussing the necessity for continued investigations, a path is envisioned toward enhancing comprehension of human performance and, in turn, advancing development of personalized medicine.

## 2 Physiological differences

Through the scope of anatomy, numerous variations have been noted in the cardiovascular system between males and females. Male hearts have been shown to possess higher left ventricular (LV) mass, and cardiac output compared to females. On the contrary, female hearts show increased ejection fraction and heart rate comparatively. They also tend to display higher stiffness and host a higher population of ventricular cardiomyocytes than their male counterparts (8), which may facilitate better preservation of myocardial mass observed in women as they age (9). Furthermore, men have larger blood vessels, while women tend to have lower blood pressure but a faster resting heart rate. These changes scratch the surface of disparities between men and women when it comes to risk, phenotype, and harms during CVD progression (Table 1).

**Table 1:** Main cardiac physiological differences between males and females.

Cardiac Feature	Male	Female	Citations
Heart size	Higher	Lower	(2, 3)
Cardiac output	Higher	Lower	(10, 11)
Heart rate	Lower	Higher	(12, 13)
LV and RV ejection fraction	Lower	Higher	(14, 15)
Cardiomyocyte number	Lower	Higher	(8)
Stiffness	Lower	Higher	(16, 17)
Blood pressure	Higher	Lower	(18, 19)
Blood vessels	Larger	Smaller	(20, 21)
Repolarization	Shorter and Faster	Longer and Slower	(22, 23)
QT intervals	Shorter	Longer	(12, 24)

Various risk factors associated with development of cardiac disease also underscore notable disparities as women exhibit heightened susceptibility to many of these effects. Notably, diabetes mellitus in women carries more than twice the likelihood of culminating in cardiac disease compared to its manifestation in men (25, 26). Hypertension poses a greater risk of leading to the induction of heart failure in women (27, 28), alongside obesity which is more pronounced among women (29), and further increases risk of cardiovascular pathologies for this gender (30). Smoking also prompts a higher factor of risk for females (31, 32), though this risk is attenuated demographically by a lesser population of female smokers comparatively (33). A recent increase in smoking rates among the youth, particularly young women (34), gives rise to significant alarm as this is further associated with significantly increased risks for peripartum cardiomyopathy (35).

Lifestyle impacts demonstrate significant provenance as dietary choices, drug abuse and alcohol consumption emerge as pivotal influencers of heart failure risk. A dietary pattern characterized by inclusion of fried foods, processed meats, eggs, added fats, and sugary beverages, often adopted by African American women, substantially amplifies vulnerability to heart failure (36). While alcohol consumption is more prevalent among men, its adverse cardio-pathological consequences disproportionately impact women (37). Socio-economic factors encompassing income inequality, stress, and depression also assume an elevated significance in the incidence of cardiac pathology among women (38–41). Of particular focus is Takotsubo cardiomyopathy, a cardiac condition triggered by emotional or physical stress, which logically displays a disproportionately higher occurrence in women (42, 43).

To further add insult to injury, there are specific risk factors and cardiac diseases unique to women. Risk of peripartum cardiomyopathy and subsequent heart failure incidents post-pregnancy is significantly increased by conditions such as gestational diabetes and hypertensive disorders linked to pregnancy, including but not limited to preeclampsia and eclampsia (44, 45). Therapies for breast cancer, the most prevalent cancer in women, have been associated with elevated incidences of heart failure (46). Furthermore, due to physiological demands of menstrual cycles, pregnancy, and childbirth, susceptibility to both anemia and iron deficiency are drastically increased, which have both been shown to contribute to progression of cardiac diseases (47).

Despite the comparable lifelong susceptibility to CVD across genders, women generally confront the onset of CVD at later life stages, with augmented incidence post-menopause. Pre-menopausal women demonstrate a lower risk of myocardial infarction (MI) events compared to men (48). Additionally, females have exhibited superior survival and recovery in response to permanent coronary ligation (49). When it comes to cardiac disease, men commonly develop heart failure with reduced ejection fraction (HFrEF), while women are more inclined to experience heart failure with preserved ejection fraction (HFpEF) (50, 51). Men tend to experience complications related to macrovascular issues associated with plaque rupture in larger arteries, whereas women often present with obstructive diseases targeting microvasculature or non-ischemic causes primarily resulting from hypertension (28, 52–54). These microvascular targeting diseases often result from inflammatory or autoimmune disorders in women. While men show no elevated prevalence of plaque rupture, vulnerability of these structures increases with age in women (55, 56). Arrhythmias are more frequently seen in men, often linked to intense effort and exercise (57).

Upon examining hypertrophic patterns, distinct gender-based observations arise. Women tend to develop a concentric heart remodeling characterized by smaller ventricular dilation. This is attributed to progression from arterial hypertension to cardiac disease. Conversely, men tend to display eccentric remodeling, which is associated with coronary artery disease and mainly caused by plaque buildup (58).

Discrepancies in heart failure symptoms between men and women have also been observed. While chest pressure is a common symptom for both, women experience additional non-chest-related symptoms, including nausea, dizziness, fatigue, as well as pain between the shoulder blades, abdomen, or back. These disparities might be attributed to the fact that women have a higher likelihood of developing coronary microvascular disease, in contrast to men, who tend to experience blockages in larger arteries.

## 3 Sex chromosomes

The presence of a single unique X chromosome in males leads to the need for dosage compensation. This is executed through an epigenetic mechanism for balancing female gene expression, known as X chromosome inactivation (XCI). This intricate process is orchestrated by epigenetic factors such as DNA methylation, chromatin structural changes, and histone marks. During embryonic development, one of the two copies of the X chromosome is condensed by the long non-coding RNA XIST. The crucial aspect of this process relies on the methylation status of the 5’ end of the *Xist* gene, which becomes unmethylated in the inactivated X chromosome while remaining methylated in the active X chromosome (59). The presence of XIST initiates the assembly of a series of polycistronic complexes that drive DNA methylation and the progression of heterochromatin formation along the entire chromosome, effectively silencing gene expression (60). XCI also promotes formation of two chromatin structural super-domains delineated by the region encoding DXZ4 long non-coding RNA (61). This high-order structural reorganization of the chromosome is mediated through interaction of the *Dxz4*/DXZ4 loci and chromatin organizer CTCF, underscoring the significance of DXZ4 in configuring X chromosome inactivation (62). Another X-linked long non-coding RNA crucial for this process is FIRRE. This participates in XCI by interacting with chromatin organizers and the nuclear matrix to facilitate positioning of the inactive X chromosome close to repressive locations, such as the perinucleolar region and nuclear periphery (63).

**Figure 2:**
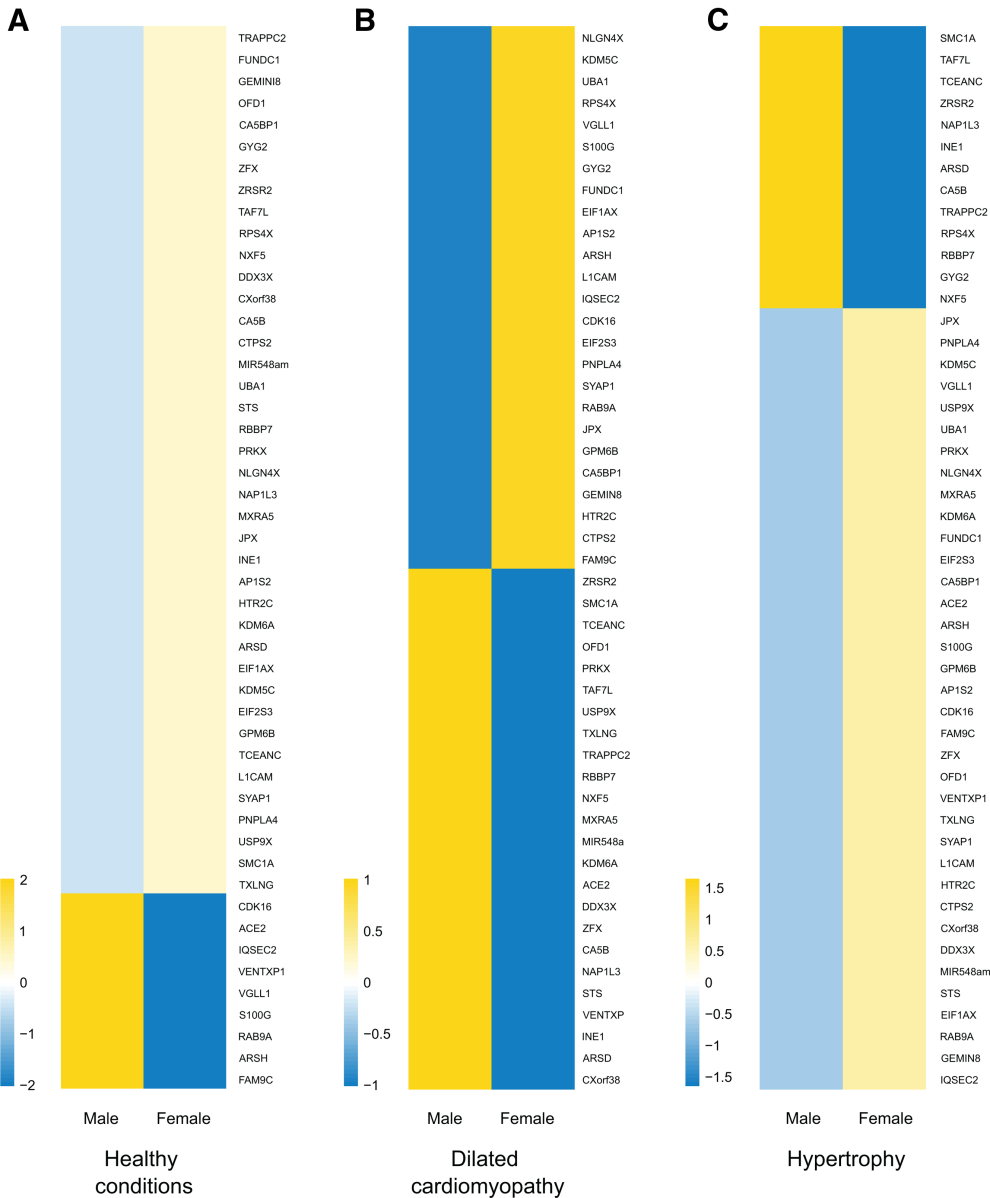
X-escapee gene expression in cardiac disease. (**A**) Heatmap displaying the normalized read counts of the scape gene when comparing healthy male and female patients. Under healthy conditions, most X-escapee genes exhibit higher expression levels in females compared to males. (**B**) Heatmap showing log2(healthy/sick) for escape genes in male and female patients with dilated cardiomyopathy. (**C**) Similar analysis to B but comparing patients with hypertrophy. The data obtained from pathological samples show clear gene expression differences between sexes, suggesting that escape genes significantly impact the disease’s gender-specific progression.

Despite transcriptional suppression mechanisms aimed at achieving XCI, a subset of X genes, approximately 3% in mice and 15% in humans, manage to evade this transformation (64, 65). These “escape genes” introduce a degree of differential gene expression between the sexes, potentially contributing to sex-related variations in physiological traits and susceptibility to diseases (66). In an exhaustive compilation of escape genes listed in a previous study (67), we successfully identified genes linked to cardiac pathology and others involved in regulation of gene expression, some through epigenetic mechanisms (Table 2). Intriguingly, when we study the transcriptional behavior of the entire list of escape genes comparing healthy and sick patients afflicted with hypertrophy or dilated cardiomyopathy (85) we observed a very distinctive pattern when comparing both sexes (Figure 2). This finding confirms the participation of escape genes such as *Kdm5*, *Kdm6*, *Ace2* and *Uba1* in sculpting cardiac pathological phenotypes and strongly implies that genes previously unrelated with cardiac pathology also participate in the onset and progression of the disease. The pronounced disparity in expression patterns observed between male and female subjects also underscores the capacity that these genes have to instigate discernible differences in the pathology between both sexes. While further investigation is necessary to validate and enhance significance of these results, it is hoped that these findings will provide valuable insights for future research.

**Table 2:** List of X-escapee genes associated with epigenetic processes and/or cardiac function.

Escape Gene	Association with epigenetic processes and/or cardiac disease	Citations
*Mxra5*	Elevated expression observed in the later stages of heart failure and linked to aortic stenosis	(68, 69)
*Ofd1*	Mutations in this gene are associated with congenital heart defects	(70)
*Ace2*	Provides a protective role in the context of myocardial infarction	(71)
*Fundc1*	Cardiac dysfunction results from its deficiency	(72)
*Jpx*	Long non-coding RNA exhibiting a cardioprotective role against acute myocardial I/R injury	(73)
*Rbbp7*	Engages in remodeling chromatin structure and in the recruitment of epigenetic complexes	(74, 75)
*Usp9x*	Stabilizes the Polycomb Repressive Complex 2, contributing to its association with heart defects	(76, 77)
*Zfx*	Operates as a DNA-binding transcriptional activator	(78)
*Kdm6a*	Histone demethylase that participate in the transcriptional regulation of autosomal genes, encompassing several specific to the heart	(79)
*Kdm5c*		
*Uba1*	Closely linked to cardiac remodeling and heart disease	(80)
*Smc1*	Its mutation can lead to congenital cardiac defects (Cornelia de Lange syndrome)	(81)
*Nap1l3*	Participates in the epigenetic regulation of gene expression. Its levels are increased in the left ventricle of patients with cardiac pathology	(82)
*Eif2s3xI*	Controls gene expression related to sex differences in response to myocardial I/R injury	(83)
*Phf8*	Histone demethylase able to attenuate cardiac hypertrophy under conditions of cardiac overload	(84)

Disparities in hormone levels between males and females play a crucial role in divergent manifestations of cardiovascular disease across genders. As the distinct hormonal landscape stabilizes early in life, disentangling the respective contributions of sex chromosomes and gonadal hormones to sex differences has been a challenging task. To address this challenge, the four core genotypes mouse model (86) (FCG) was developed. This model encompasses the creation of mice with four distinct combinations featuring XX and XY gonadal males alongside XX and XY gonadal females. This design facilitates the examination of the effects of sex chromosomes independently from gonadal sex and vice versa. Studies with these mice have uncovered that vulnerability to myocardial ischemia-reperfusion (I/R) injury escalates with the number of X chromosomes (83). Intriguingly, expression levels of several specific escape genes (*Eif2s3x*, *Kdm5c*, *Kdm6a*, and *Usp9x*) were elevated in the heart tissue of XX mice compared to that of their XY counterparts. This implies a potential involvement of these genes in mediating the observed differences in response to I/R. The same group used the FCG model to demonstrate that the presence of a Y chromosome confers protection against the development of pulmonary hypertension, a cardiac disease with a higher prevalence in females than males (87). Transcriptional analyses suggest that genes encoded in the Y chromosome and expressed in the heart and lungs (*Ddx3y*, *Eif2s3y*, *Kdm5d*, and *Uty*) may impact this effect through effects on proliferation, apoptosis, and epigenetic regulation (88). This model has also been instrumental in demonstrating a correlation between the number of X chromosomes and an increase in body weight, a factor that appears to be independent of the presence of Y chromosomes (89). This correlation held true when comparing mice with one X chromosome to those with two (90), indicating that X chromosome dosage may pose a risk for obesity. Consequently, this association with obesity suggests a potential link to cardiac disease, where XCI escape genes potentially underlie observed phenotypes.

## 4 Genetic differences

Genetic variations, often presented as mutations, wield a notable influence on the likelihood of developing heart disease. Some of these variations demonstrate a sex-biased impact, meaning they differentially affect the probabilities of heart disease development in a sex-specific manner.

### 4.1 X-linked genetic variants

Genetic variations on the X chromosome, known as X-linked genetic variants, play a significant role in development of cardiac disease phenotypes. Three clear examples of pathologies with cardiac implications associated with variations on the X chromosome are Danon disease, Duchenne muscular dystrophy and Fabry disease. In the case of Danon disease, mutation of the *Lamp2* gene promotes the accumulation of autophagic vacuoles, a critical process in the onset of the disease (91). The presence of only one X chromosome in males expedites the start of this disease, accelerating the progression toward heart failure when compared to females (92). Duchenne muscular dystrophy stems from mutation of the *Dmd* gene, responsible for encoding dystrophin. This progressive disorder contributes to breathing difficulties and cardiomyopathies, which are less pronounced in females due to their compensatory second X chromosome (93). Fabry disease is attributed to the accumulation of glycosphingolipids, often due to mutation of the alpha-galactosidase A gene (*Gla*). This disorder leads to cardiac disease, renal failure, and early mortality (94). Akin to Danon and Duchenne diseases, the impact of Fabry pathology is more pronounced in males due to X hemizygosity, although heterozygous women may exhibit similar symptoms due to defective X chromosome inactivation (95).

### 4.2 Autosomal gene variants

Genetic alterations in autosomal genes also hold a crucial role in sex differences observed within cardiac diseases such as dilated cardiomyopathy, congestive heart failure and cardiac hypertrophy. Truncating mutations in the Ttn gene, responsible for encoding TITIN, a vital sarcomere component, and the Dsp gene, encoding Desmoplakin, both significantly impact the onset of dilated cardiomyopathy (DCM). Males carrying one of the Ttn truncating variants exhibit a higher risk of DCM development compared to females, resulting in worsened systolic function and elevated risk of atrial fibrillation (96). In contrast, women with either of these variants are more likely to develop peripartum DCM (97). Truncating variants in the Dsp gene exhibit higher penetrance in women (98), with relatively similar phenotypic effects between sexes. Variants in the Lmna gene, responsible for encoding nuclear lamina proteins A and C, also contribute to DCM development. Notably, female carriers tend to manifest clinical symptoms at a later age (99) and their effects are also associated with lower incidence of life-threatening ventricular tachyarrhythmia (100). Cardiomyopathies linked to mutations in the Flnc gene, encoding Filamin C, an actin-binding protein crucial for sarcomeric stability, pose a higher risk of cardiovascular pathology in men (101). Alterations to the Ttr gene induce misfolding, aggregation, and deposition of the transthyretin protein. This leads to the formation of amyloid fibrils within the myocardium, which promotes cardiac amyloidosis, associated with congestive heart failure and irregular heart rhythm (102), that show a higher predominance in men (103, 104). With regards to hypertrophic cardiomyopathy, women exhibit a greater propensity for sarcomere mutations, specifically in Mybpc3, Myh7, and thin filament genes. Furthermore, it is noteworthy that while the onset of disease occurs later in women, they face an elevated risk of developing heart failure or death (105). These observations also suggest that women may require a higher number of genetic alterations to develop heart failure, implying an increased likelihood of transmitting the disease to their offspring (106).

## 5 Epigenetic differences

The field of epigenetics studies all changes in DNA that affect gene expression and can be inherited without involving the alteration of the underlying sequence (107). A variety of factors, such as environmental conditions, lifestyle choices, and developmental processes can influence these modifications. Epigenetic changes can activate or repress genes, impacting an individual’s phenotype or cellular functions. Key mechanisms of epigenetics include DNA methylation, histone modifications and chromatin structural modifications. A comprehensive understanding of epigenetics is imperative for unraveling the intricate regulation of genes between males and females, as well as comprehending how external factors shape an individual’s health and susceptibility to diseases. When exploring the existing literature on how epigenetics contributes to sex differences in cardiac pathology, a noticeable inclination towards investigating DNA methylation is observed. This preference likely arises from practical advantages of using blood samples over biopsies in human patients, along with relatively straightforward computational requirements for data analysis in these experiments.

### 5.1 DNA methylation

DNA methylation involves a chemical modification of DNA wherein methyl groups are added to cytosine nucleotides, predominantly occurring at CpG dinucleotides. Genomic regions abundant in CpG dinucleotides are termed CpG islands and are commonly situated in proximity to gene promoters. DNA methylation can act as a regulatory switch for gene expression, with methylated CpG sites often associated with gene repression or silencing. The methylation status of various genes is widely recognized for significant involvement in development of cardiovascular disease alongside a clear potential to serve as a pathological diagnostic biomarker (108). Additionally, it is well-established that disparities in DNA methylation between sexes influence gene expression by means of epigenetic mechanisms (109). However, research focused on investigating sex-specific cardiac DNA methylation differences remains limited, primarily due to a predominant focus on male samples in past studies (110, 111).

Studies focusing on examination of variations in DNA methylation levels between genders delve into relationships between DNA methylation and various factors, including diverse cardiovascular diseases, environmental factors, and associated pathological risk factors. One interesting finding at the environmental level involves the impact of lead exposure during murine gestation and lactation, including significant sex-specific cardiac DNA methylation alterations in adulthood (112). In this work, analysis of regions exhibiting differential methylation in males has revealed associations with Notch and Hedgehog pathways, vital in cardiac development and emergence of cardiac pathologies, respectively. In females, identified loci have been linked to lysine demethylation and arginine hydroxylation pathways, crucial for epigenetic processes regulating cardiac chromatin structure and heart function. Furthermore, this research unearthed various cardiovascular-related genes with differing expression and/or methylation status (*Galnt2*, *Atg5*, *Ank2*, *Cux1*, *Lamp2*, *Akap1*, and *Grk5*) across males and females. This discovery suggests that modifications to these genes are important in influencing susceptibility to cardiac pathological outcomes following environmental lead exposure.

One study of DNA methylation in blood samples from ischemic stroke patients has revealed intriguing gender differences. Global methylation levels were lower in men than in their counterpart (113). Further investigation showed that when compared to healthy controls, male patients who had undergone ischemic stroke carried lower methylation levels in the *Mmp2* gene, encoding a matrix metalloproteinase that plays a role in the recovery processes after injury (114). Another noteworthy finding from the study of these patients was the significant association between methylation of the *Pla2g7* promoter and risk of developing coronary heart disease in women but not in men (115). The *Pla2g7* gene encodes lipoprotein-associated phospholipase A2, which accumulates in atherosclerotic plaques and is a predictive biomarker for atherosclerotic diseases (116). Moreover, patients with elevated PTX3 levels, a member of the pentraxin superfamily that is associated with atherosclerotic lesions, face increased cardiac event risks. Reduced methylation of the *Ptx3* promoter is linked to increases in PTX3 plasma levels during coronary artery disease. Interestingly, these lessened methylation levels have been linked to higher neutrophil-to-lymphocyte ratios in men but not women. This suggests that inflammation plays a role in observed disparities between sexes during the formation of atherosclerotic plaques (117).

In the context of risk associated with developing cardiac disease, a quantitative mass spectrometry experiment of peripheral blood revealed that methylation of the *Actb* gene, which encodes the cytoskeletal protein *β*-actin, is strongly associated with risk of coronary heart disease when comparing sexes (118). Myocardial infarction (MI) risk in women has been associated with DNA methylation of *Ins* and *Gnasas* loci. These genes encode for insulin and the alpha-subunit of the stimulatory G protein, respectively and are responsible for regulating fetal growth. Methylation at these loci is more prevalent among women than men in MI cases compared to healthy controls (119). Through MALDI-TOF mass spectrometry experiments conducted on whole blood samples, it was discovered that reduced methylation of the *F2rl3* gene, responsible for encoding protease-activated receptor-4 (a crucial factor mediating human platelet activation that also serves as a smoking biomarker), is closely linked to mortality associated with cardiac disease. Interestingly, this association exhibits greater strength among men (120). A study investigating links between body mass index (BMI) and differential methylation through whole-blood methylation analysis identified a particularly robust association in men. This was localized to a specific CpG site near the *Lgals3bp* gene (121), a recognized marker for obesity and metabolic syndrome (122). Additionally, higher levels of *Line-1* methylation, responsible for coding transposable elements, were detected in men using peripheral blood. Elevated *Line-1* methylation levels are linked to increased levels of low-density lipoprotein (LDL) and reduced levels of high-density lipoprotein (HDL), well-established risk factors for atherosclerosis. Interestingly, the same study found a significant association between *Line-1* methylation and BMI in females (123). Implementing the MEpiTYPER mass-spectroscopy technique to analyze genomic DNA, it was demonstrated that methylation in several CpGs on the *Fabp3* gene, responsible for encoding fatty acid binding protein 3, a regulator of fatty acid solubility and mobility, is sex-dependent (124). This research also highlighted that methylation of these CpGs is strongly associated with plasma cholesterol levels, potentially playing a distinct role in cardiovascular disease development between males and females.

### 5.2 Histone modifications

Histone modifications, also known as histone marks, are chemical changes to histone proteins that help DNA package. Mediated by histone modifiers, these marks include acetylation, methylation, phosphorylation, ubiquitination, and sumoylation, among others (125). Each modification alters the structure and accessibility of chromatin, influencing gene expression. Acetylation typically promotes gene activation by loosening chromatin, while methylation can lead to either gene activation or repression, depending on location. Phosphorylation, ubiquitination, and sumoylation have specific roles in processes such as DNA repair and transcriptional regulation. Histone marks are essential for fine-tuned regulation of cellular processes, allowing genes to be appropriately activated or repressed in response to various signals and necessities. In the cardiovascular field, the role of histone marks and histone modifiers have been extensively explored due to their essential involvement in processes related to development and treatment of cardiovascular diseases (108, 126). However, investigation of sex-based differences within this context remains somewhat limited, primarily focusing on activity of histone deacetylases. A study comparing male and female responses to myocardial infarction revealed that absence of histone deacetylases 5 and 9 provides protection to females. This is accomplished through mitigation of maladaptive remodeling, partially due to activation of neoangiogenesis (127). A different work used estrogens or estrogen receptor agonists to inhibit production of pro-hypertrophic class I histone deacetylases (e.g., HDAC2) and to counteract effects of anti-hypertrophic class II histone deacetylases (HDAC4 and 5) that are promoted by angiotensin II (128). Estrogen therapy has been proven effective in preventing cardiac dysfunction associated with post-menopausal metabolic syndrome. It achieves this by restoring expression of *Sirt1,* a NAD-dependent deacetylase, and suppressing acetylation of histone 3, which is induced by angiotensin II (129).

**Figure 3:**
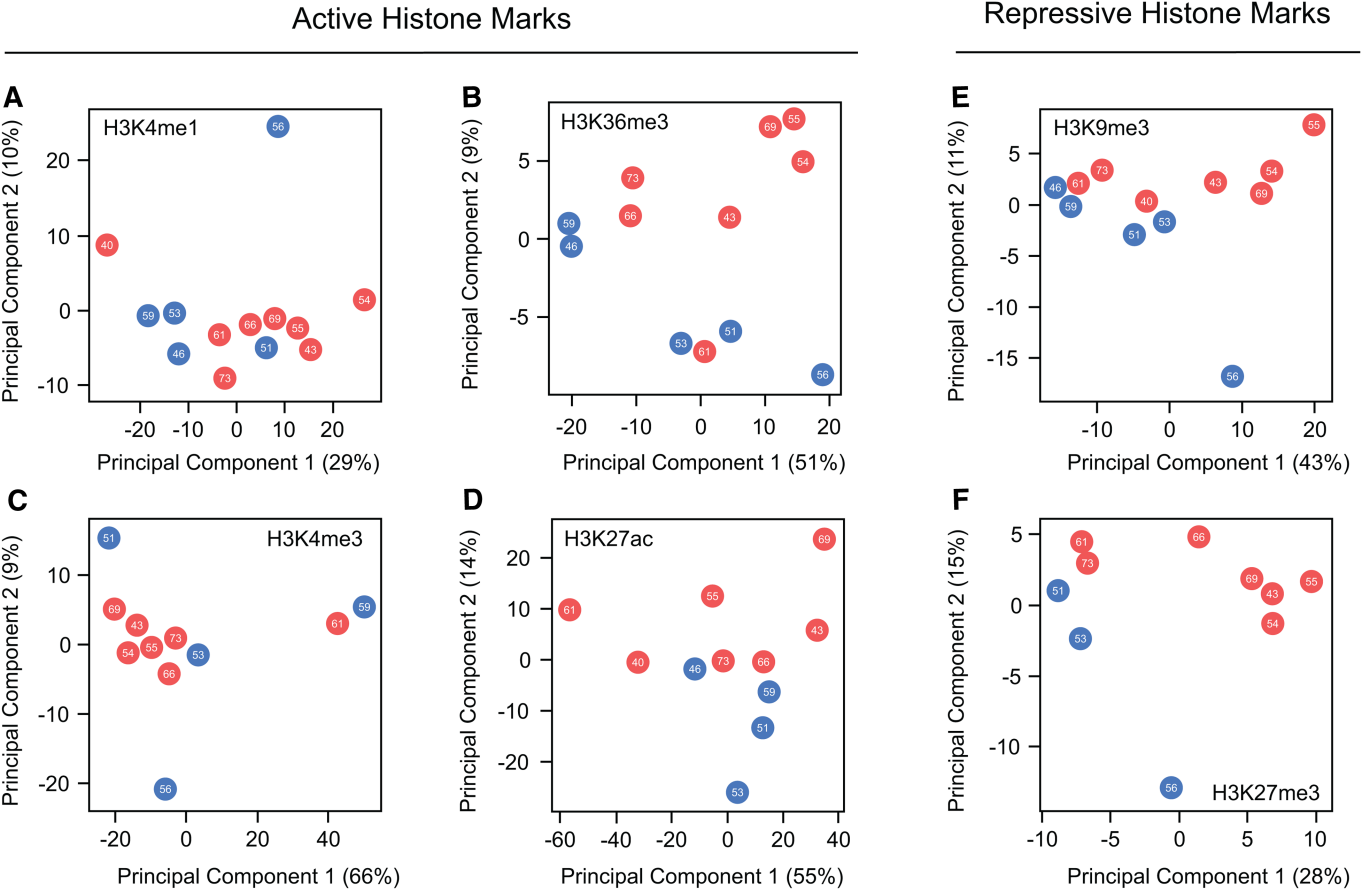
Global comparison of histone marks deposition in healthy male and female hearts. (**A–D**) Principal component analysis comparing ChIP-seq peaks from the hearts of males and females across various ages for the active histone marks, H3K4me1, H3K36me3, H3K4me3 and H3K27ac. (**E,F**) Parallel PCA analysis to (**A–D**), specifically examining the repressive marks H3K9me3 and H3K27me3. Data demonstrate no discernible differences in the global distribution of marks along the genome between the sexes. This suggests that the changes influencing variations in gene expression are situated at a locus level. Each dot represents a different patient, with red dots representing females and blue denoting males. The number within each dot indicates age of the patient.

While these findings suggest that beneficial effects of estrogens in preventing cardiac pathology are partially linked to reorganization of histone mark profiles, none of the mentioned articles have extensively explored the nature of these changes or their precise locations. To explore potential differences in histone mark deposition between healthy adult male and female human hearts at different ages, we conducted principal component analyses (PCA) using published data generated by the ENCODE project (130). Our work, which compared ChIP-seq peaks from active marks (H3K4me1, H3K27ac, H3K36me3, and H4me3) and repressive histone marks (H3K9me3 and H3K27me3), reveals no clear separation in the global distribution of these marks between sexes or based on age (Figure 3). These results clearly show high similarity in the global histone landscape of males and females and suggest that the sex-differences in gene expression mediated by these marks are linked to local specific alterations. These findings also underscore the crucial point that minor adjustments in epigenetic processes can exert a substantial effect on gene expression, elucidating observed differences in physical traits. The question arising after this analysis is whether the onset of heart failure correlates with sex-specific global or local shifts in histone mark deposition that influence the expression of genes pivotal in the development of the cardiac pathological phenotype. Exploring this connection warrants attention in future research, and we look forward to drawing forth inspiration towards filling the knowledge gaps outlined in this review.

### 5.3 Chromatin structure

Chromatin structure includes both local and high-order organization. At the local level, chromatin structure involves arrangement of DNA around histone proteins to form nucleosomes. The tightness or looseness of this packaging influences gene accessibility. Loosely packed nucleosomes allow for easier access to DNA, mostly enabling gene expression. In contrast, tightly packed nucleosomes restrict access, usually silencing gene expression. Chemical modification to histones, such as acetylation or methylation, influences local chromatin structures and serves as “epigenetic tags” that help to determine how tightly or loosely DNA is wrapped around nucleosomes. High-order chromatin structure involves organization of entire chromosomes within nuclei. This organization is far from random and encompasses different hierarchical layers of 3D chromatin architecture. From the top to the bottom in the topological organization of the genome chromatin architecture is organized in epigenetic environments termed A and B compartments, regions with high levels of intra-domain interactions known as topologically associated domains (TADs) and local interactions mediated by chromatin loops (131). These structural features orchestrate gene localization within the nucleus and determine which genes are brought into proximity, facilitating gene activation, isolation, transcriptional co-regulation and interaction with regulatory elements such as enhancers. Several factors wield significant influence in shaping the three-dimensional architecture of the genome. Among them, CTCF and cohesin complexes stand out for their ability to work synergistically, creating and stabilizing chromatin loops and TADs (132, 133). This intricate process ensures that the correct genes are properly positioned for coordinated regulation, impacting the overall gene expression landscape and, consequently, observed phenotype.

Organization of cardiac chromatin operates at both local and high-order scales, playing a crucial role in the proper functioning of the heart and the development of cardiac pathologies. Local chromatin organization, facilitated by chromatin remodelers such as the SWI/SNF complex, has previously been linked to cardiovascular function and pathology (134). Bulk ATAC-seq experiments have demonstrated that chromatin remodeling precedes cardiac disease, highlighting the potential of chromatin structural features as valuable biomarkers and potential targets for future clinical interventions (135). Furthermore, the application of single-cell ATAC-seq in the heart has allowed for the characterization of distinct cell states and subtypes, including cardiomyocytes, endothelial cells, fibroblasts, and myeloid cells, each associated with different gene expressions and disease conditions when comparing healthy and diseased patients (136). Single-nucleus ATAC-seq has also proven instrumental in identifying cis-regulatory elements involved in governing gene expression and the gene-regulatory networks associated with the control of both healthy and pathological heart phenotypes (137).

High-order chromatin structure in the heart has been investigated through Hi-C studies, particularly in mouse adult cardiomyocytes. These works performed in adult mouse cardiomyocytes showed that induction of heart failure promoted by depletion of CTCF is mediated by chromatin structural changes that alter cardiac gene expression. Notably, many of these topological changes were also identified in a model of pressure overload induced by transverse aortic constriction (TAC), suggesting that global chromatin reorganization associated with cardiac disease shares significant similarities irrespective of the underlying cause of the pathology (138, 139). Interaction between distal regulatory elements and their target genes is a specific mechanism directly linked to the control of the transcriptional landscape associated with cardiac disease on a large-scale. Disruption of a long-range looping that communicates the locus encoding the transcription factor PITX2 with a regulatory element located upstream of the gene specifically reduces its expression in males (140). This, in turn, increases their susceptibility to developing atrial fibrillation, likely due to the shortening of the atrial action potential mediated by transcriptional dysregulation of genes coding for potassium and calcium channels (141). Other examples where altering the enhancer-gene interaction is associated with the cardiac phenotype include the enhancer mediating the switch in expression from MYH6 to MYH7 (142) and the one coordinating the expression of the *Nppa* and *Nppb* genes (143). In the first case, disruption of the enhancer prevents the shift in the MYH6/7 ratio associated with the development of heart failure, suggesting that eliminating this interaction has a potential role in preventing cardiac pathology (144). In the second case, a total lack of communication between the regulatory region and the *Nppa* and *Nppb* genes, both implicated in the response to cardiac injury and stress, promotes cardiac hypertrophy.

**Figure 4:**
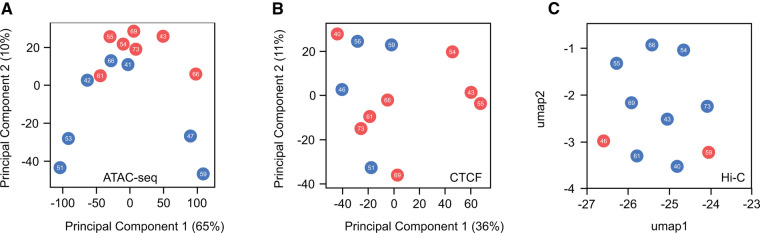
Global comparison of chromatin accessibility and high-order chromatin structure in healthy male and female hearts. (**A**) Principal component analysis (PCA) of ATAC-seq peaks indicates no distinct separation between male and female samples regarding sex or age. (**B**) Similarly, PCA analysis of CTCF ChIP-seq peaks, a master regulator of chromatin structure, reveals no discernible differences. (**C**) Uniform Manifold Approximation and Projection (UMAP) plot of Hi-C interactions shows no variations in higher-order chromatin structure between sexes. These findings suggest a high similarity in the global organization of chromatin when comparing males and females. A more in-depth investigation of these features is needed to identify whether local changes contribute to differences in cardiac disease susceptibility between the sexes.

Similar to challenges faced in the study of histone marks, understanding of structural differences related to gender remains constrained. This limitation is partially due to the resource-intensive and laborious nature of the chromatin conformation capture (3C, 4C, Hi-C, SPRITE, GAM) and assay for transposase accessible (ATAC) protocols. These approaches require extensive sequencing coverage to effectively identify and investigate different chromatin features, posing challenges to exploration of sex-based disparities at the structural level. While this field is currently expanding, a comprehensive global analysis of sex-based distinctions has yet to be performed. To shed light on differences in chromatin structure between males and females at both local and high-order levels, we conducted a comparative analysis using human heart data accessible through the ENCODE project (Figure 4). This analysis examines chromatin accessibility (ATAC-seq), 3D structure of the genome (Hi-C) and CTCF expression (CTCF ChIP-seq). As previously seen, when histone marks were analyzed, no discernible differences emerged between sexes or concerning age. These findings suggest that, at a global scale, chromatin organization remains highly similar between males and females under healthy conditions, implying that any disparities between sexes are localized to specific genomic loci. The available data for this analysis proved insufficient to determine if sex-based disparities are present during disease progression or presentation, as all samples were of healthy individuals. This leads to the important question as to whether the structural changes observed during the development of heart disease (138) exhibit gender-specific variations and whether these potential distinctions contribute to transcriptional alterations, ultimately manifesting in diverse cardiac pathological outcomes observed between males and females.

## 6 Discussion

Cardiovascular disease, a leading cause of morbidity and mortality in both genders, displays notable variations in presentation, progression, and impact between males and females. While the literature on cardiac sex differences continues to grow, the field’s current knowledge predominantly centers on comprehending physiological effects driven by variations in hormone levels. Thanks to the substantial research efforts from the allocation of institutional and academic resources to investigate the mechanisms underlying sex-related cardiac differences, the inclusion of females in genetic and epigenetic studies is beginning to emerge. Throughout this review, we have highlighted the current knowledge on how X chromosome inactivation escape genes, genetic variants and epigenetic factors such as DNA methylation, histone marks, and chromatin structure mediate the cardiac differences observed between males and females. In this context, it is worth noting that while DNA methylation has received significant attention in the context of sex-specific disparities, other epigenetic factors have yet to be thoroughly investigated.

Recent research (145) has brought to light a notable revelation: a significant portion of sex-biased genes are not randomly scattered throughout the genome. Instead, these genes are clustered within specific genomic regions, suggesting that a coordinated activity of epigenetic mechanisms is in control of their expression. This discovery emphasizes the importance of ongoing investigation into epigenetic mechanisms, which can be influenced by environmental and lifestyle factors. In an effort to stimulate investigation in this direction, we conducted various analyses focused on the differential expression of escape genes during the development of cardiac pathology and the global distribution of different epigenetic effects when comparing males and females. This data has brought to light two significant findings: 1) a remarkable divergence in the pathological transcriptional behavior of the escape genes between sexes and 2) the absence of differences in the global distribution of histone marks, organization of chromatin remodeling and high-order chromatin structure and CTCF binding when comparing male and female healthy hearts. These insights suggest that genes evading X-chromosome inactivation contribute to the development of cardiac pathology, and the observed epigenetic effects influencing sex-based gene differences in the heart appear to operate at a local scale. Although aging is commonly acknowledged as a factor contributing to cardiac phenotypic distinctions between sexes (146), our findings revealed no apparent variations in any epigenetic factors associated with age. A potential limitation that could explain the lack of differences is the limited range of sample ages and relatively small size of the heart samples within the ENCODE project.

As the scientific understanding of how epigenetic processes differentially influence the onset and progression of cardiac disease in males and females continues to evolve, new inquiries emerge on the horizon. For instance, are there local variations in histone marks and chromatin structure contributing to sex-related disparities in cardiac phenotypes? Can the detected alterations in high-order chromatin structure during development and progression of cardiac diseases distinctly impact males and females? Do specific loci or regions in the genome exist where sex-biased genes and epigenetic mechanisms interact to regulate cardiac gene expression? How do environmental and lifestyle factors mediate sex-specific differences in epigenetic patterns related to cardiac health? Can the study of epigenetic modifications provide insights into personalized treatments for cardiovascular diseases based on an individual’s sex? What potential therapeutic interventions can be developed by targeting epigenetic mechanisms to address sex-related disparities in cardiovascular health? Furthermore, how might cross-hormone therapy impact epigenetic mechanisms in the context of cardiac health for transgender individuals?

To address these inquiries comprehensively, dedicated studies aimed at unveiling cardiac epigenetic differences between males and females should be conducted, rather than treating them as supplementary to studies focused on identifying physiological differences. Ideally, a robust dataset would include both clinical data and biological samples from a diverse and comparable cohort of male and female individuals. While blood has conventionally been a convenient source in the field due to its easy accessibility, utilizing heart tissue would provide richer insights into our context. This approach also facilitates single-cell sequencing experiments, broadening the scope of findings to different cardiac cell types. Heart tissue can be procured from various sources, such as animal models, healthy donors, biopsies, or organs unsuitable for transplant. The optimal approach would also involve studying the diverse epigenetic features using material from the same sample. Thanks to advancements in sequencing technologies associated with common epigenetic techniques (such as RNA-seq, DNA methylation profiling, ChIP-seq, ATAC-seq, and Hi-C), less starting material is now required, facilitating this requirement. Enriching the study with individuals affected by cardiac disease would also enhance our understanding, allowing us to observe how epigenetic differences evolve during the development of the pathology in both males and females. Moreover, the inclusion of transgender individuals or the Four Core Genotypes mouse model would serve to adapt the research to the new dimensions of the population and corroborate the cisgender data generated by the investigation. While this ideal research would require collaboration with multiple groups or function as part of a consortium, it is still advisable for smaller studies to incorporate as many of these recommendations as feasible.

Although much research is still required in the field, the growing inclusion of both sexes in studies, coupled with advancements in sequencing technologies and novel epigenetic protocols, is poised to empower researchers with a broader analytical scope. This progression underscores the significant promise within this field, offering insights into how sex-specific disparities can contribute to the development of tailored interventions for both males and females through personalized medicine.
